# Reusing Stadiums for a Greener Future: The Circular Design Potential of Football Architecture

**DOI:** 10.3389/fspor.2021.692632

**Published:** 2021-07-23

**Authors:** Even Smith Wergeland, Hans Kristian Hognestad

**Affiliations:** ^1^Oslo School of Architecture and Design, Oslo, Norway; ^2^Department of Outdoor-Life Studies, Sport and Physical Education, University of South-Eastern Norway, Bø, Norway

**Keywords:** sustainable architecture, circular heritage, historic stadiums, reuse & recycling of materials, football culture, maintenance

## Abstract

Since the turn of the new Millennium, there has been an increase in efforts to build environmental-friendly sports arenas around the world. Fuelled by large sporting events like the 2000 Sydney Olympics, the ‘Green Games,’ and the 2006 FIFA World Cup in Germany, stadium architecture has become a vehicle for this trend. So far, the emphasis has primarily been on new arenas, in line with the widespread belief in international architecture of the 2000s that older buildings are less energy-efficient by default. In addition to that comes a conviction that newness is needed to attract sponsors, investors, and larger audiences—a position powered by commercial interest and the idea of the stadium as an ‘urban generator.’ While new stadiums may have a significant potential when it comes to green performability, that does not necessarily mean that older stadiums are surplus to requirements, even from a climate perspective. In this paper, we look critically at the well-established strategy of replacing old stadiums with new ones by questioning the climate impact of new arenas and investigating the reuse potential of existing ones. We carry out in-depth analysis of two existing stadiums, Tynecastle Park in Edinburgh and Stadio Flaminio in Rome. One of them has already gone through renovation to remain in use while the other is vacant but currently under way to be renovated. We bring in fresh perspectives from sports science, preservation, architecture, and circular design theory to explain why older stadiums become obsolete and to challenge the premise of that destiny. The aim is not only to scrutinize the general lack of reuse but also to highlight green strategies which could give existing stadiums a longer life.

## Introduction

‘If the twentieth century can be characterized by growth or expansion, the greatest issue for the world in the twenty-first century is shrinkage’ (Hidetoshi, 2009, p. 79). According to the Japanese architect Ohno Hidetoshi, the world no longer has the capacity to absorb everything we build, produce and consume. He does not stand alone in this call for downscaling. ‘Enough: The Architecture of Degrowth’ was the heading of the 2019 Oslo Architecture Triennale. The 2021 recipients of the Pritzker Architecture Prize, one of the highest honors in the profession, were Lacaton and Vassall, the French duo whose motto is ‘Never demolish, never replace.’ Similar agendas are currently being voiced by a number of architects and planners inspired by the principles of circular economy, which is an economic system where all forms of waste are minimized through continuous use of resources (Lacy et al., 2020). This is now translating into architecture, design and heritage management as an anti-dote to overspending and waste accumulation in the building industry (Mercader-Moyano, 2017; Charter, 2018). The situation is urgent. Fresh statistics from the EU indicate that the building industry accounts for about 50% of all extracted materials in Europe and that the construction sector is responsible for over 35% of the EU's total waste generation (European Commission, 2020, p. 3.6).

The main problem, argues the architect Duncan Baker-Brown, is the *saturation* of production density, consumer goods and building mass of today's society (2017: xiv). We have been building unsustainably for so long that overspending and wastefulness has become the norm. This applies to the world of elite sports, where bigger arenas, higher standards and larger revenue has been the name of the game. Increasingly more spectacular, expensive and complex sport venues stand as symbols of what Kimberley S. Schimmel has called ‘the problematic growth model’ of sports (Schimmel, 1995, p. 145), rooted in dreams about boosterism, trickle-down economic benefits, sector expansion and capital investment.

Paradoxically, in light of these excessive tendencies, there is also much talk about sustainability in sport. But if this is supposed to become more than a rhetoric trick to land bids for sporting mega-events (Kowalska, 2017, p. 1–10), many aspects must change. The main question we are raising in this article is whether or not there is a potential within the world of sports to embrace circular thinking as an alternative form of governance. In this context we limit our attention to football architecture. What would it take, within this field of obsessive growth-orientation, to embrace degrowth as a principle for future development?

**Figure 1:**
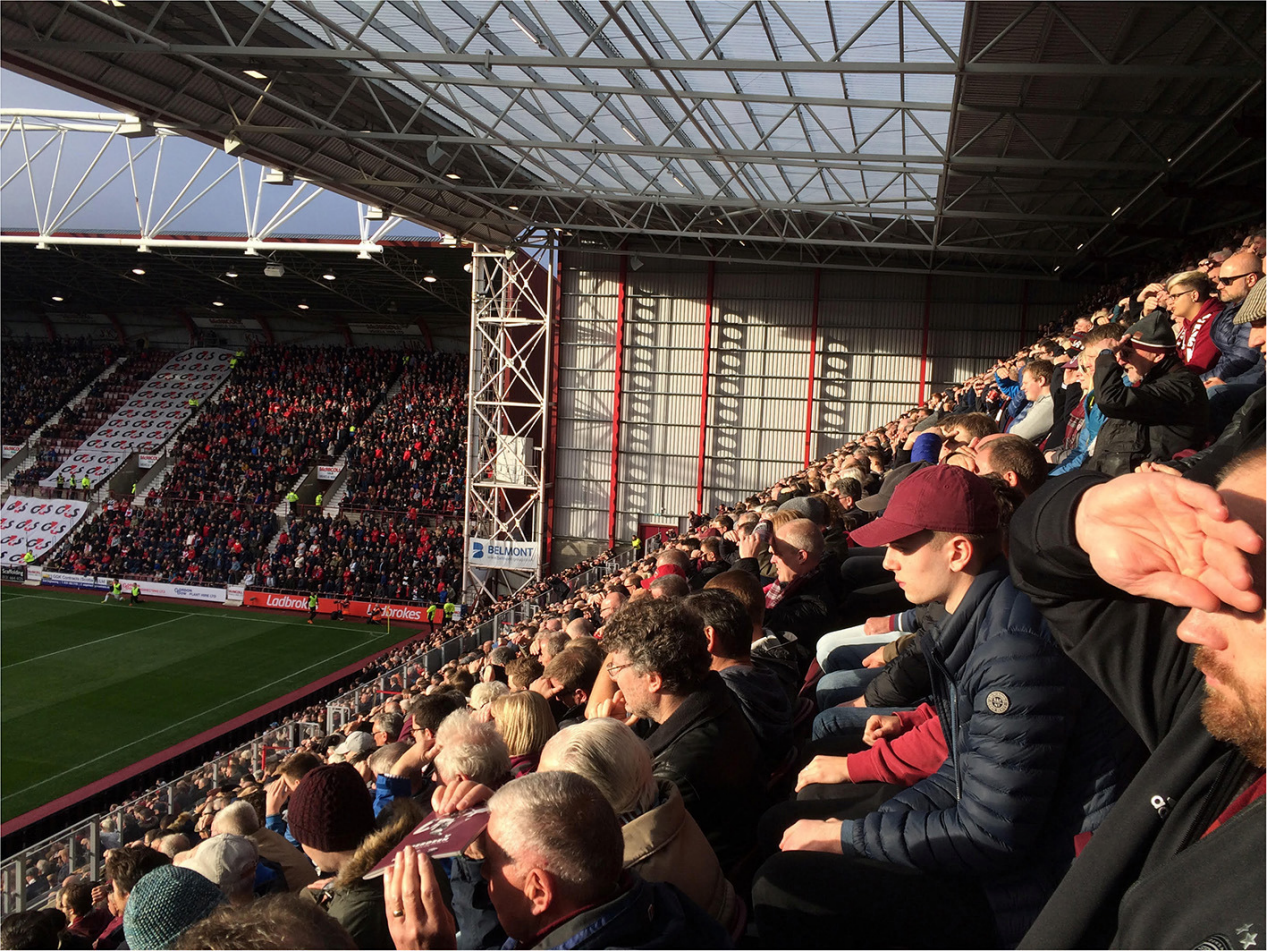
Hearts fans enjoying the sun in the new main stand at Tynecastle Park, shortly after it was opened in October 2018 (Credit: Hans K. Hognestad).

**Figure 2:**
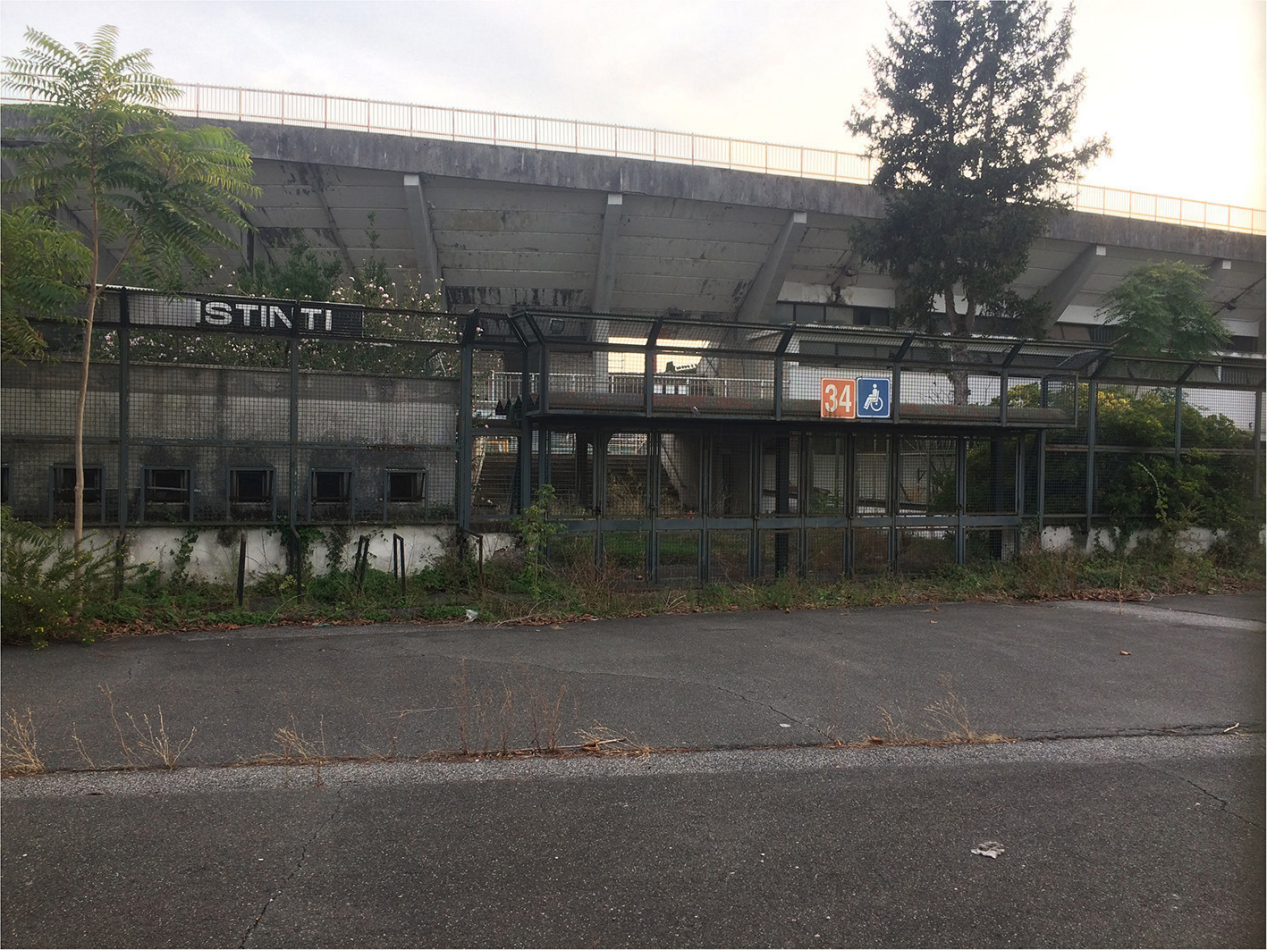
Closed and abandoned: Outside the gates of Stadio Flaminio in September 2017 (Credit: Even Smith Wergeland).

In order to discuss this we draw on three contemporary architectural preservation concepts: Adaptive reuse, maintenance architecture and circular heritage (Sample, 2016; Baker-Brown, 2017; Charter, 2018; Plevoets and van Cleempoel, 2019). Adaptive reuse and maintenance are of particular relevance to our study of two historical stadiums—Tynecastle Park (Figure 1) in Edinburgh and Stadio Flaminio (Figure 2) in Rome—and the quest of keeping them in use. Tynecastle Park was chosen as it represents an example of a socially sustainable solution and an exception to the tendency of professional football clubs moving out from inner city locations to more suburban locations, thus disrupting both social and environmental dimensions of stadium (re)construction. Stadio Flaminio is chosen due to its inner-city location and the ongoing effort to restore it under the guidance of a multi-disciplinary team of preservation experts. This process is an unusual example of a full technical and functional restoration of an historic football stadium, with the aim of re-opening it after a decade of inactivity. There is also an element of pragmatism involved in the selection. The corona pandemic has prevented us from conducting new field work, which meant that we had to rely on our previous studies. It should be noted, however, that there is not an abundance of alternatives, given the low degree of football stadium reuse in Europe.

As for key concepts, ‘Heritage’ refers to the historical values at hand, architecturally, socially and sporting-wise, while ‘circular’ comes from the aforementioned field of circular economy. The essence of circular heritage is to take an extended lifecycle perspective that focuses on maximizing value in economic, social, and cultural terms for the longest time possible (Charter, 2018). It is a protest against the urge for new things, an encouragement to reuse, repair, refurbish, remanufacture, and repurpose existing things, and a quest to recycle and recover all buildings that fall into disrepair (Foster, 2020). Our aim is to use this as a platform to cover the technical aspects of football stadium reuse alongside the issue of social sustainability—a much-neglected aspect of recent stadium design, as we argue below.

We blend these architectural preservation perspectives with critical studies of sports mega-events (Müller, 2015; Kowalska, 2017; Dendura, 2019) and studies of how the cultural practices inside and outside football stadia have been affected by globalization in the age of ‘hypercommodification’ (Giulianotti, 2002). Studies of match-day routines and other forms of supporter engagement with the stadium surroundings are of particular use in this regard (Brown, 2010; Edensor and Millington, 2010). We also draw on pioneering contributions like John Bale's ‘Playing at home’ (1991) and subsequent variations over the socio-geographical vocabulary he helped establish in the early 1990s (Bale, 1993a,b; Bale and Moen, 1995). The article backdrop also includes a selection of previous publications by the two authors of this article within these fields of knowledge (Hognestad, 2012a,b, 2017; Wergeland, 2012a,b, 2017).

## Methodological Considerations

From a methodological point of view, the article depends primarily on critical literature studies and the exchange of theoretical perspectives from several disciplines. We also base our study on several field trips to Edinburgh and Rome, conducted separately, which has provided us with field notes, photographs, and other forms of on-site documentation of the two historical stadiums in question. An important part of the data on Tynecastle Park in Edinburgh stems from an 8 months field-study of identity and meaning among supporters of Heart of Midlothian Football Club back in 1992–93, when the club eventually decided to redevelop the historic inner-city stadium rather than build a new stadium outside Edinburgh, following considerable environmental and fan activism. Data from this case study has also been drawn from several shorter field studies in more recent times, notably in 2017 and 2018 when Tynecastle Park underwent its latest redevelopments. The data on Stadio Flaminio have been collected during two field trips to Rome in the autumns of 2017 and 2018. These trips have included site visits at the stadium accompanied by professor Francesco Romeo from Sapienza Universitá di Roma. Romeo is the project leader of the ongoing process of restoring the Flaminio. He is also the director of the Nervi Virtual Lab, which carries out in-depth studies of Pier Luigi Nervi's structural systems (PLN Project, 2021). In addition to on-site guidance, Romeo lectured on the restoration project on two separate occasions at the Norwegian Institute in Rome.

The data gathered through field work, interviews, and meetings with local expertise have been balanced against additional data gained through archival research and document studies. While the ongoing pandemic stopped us from conducting further field visits, we have coordinated the substantial material we have from earlier field studies from both of these case studies, in order to write this article. We combine this thorough analysis of historical stadiums with a brief discussion of a limited selection of new stadiums, aimed at critically assessing their alleged qualities as sustainable facilities. While this evaluation remains on the surface of many complex issues and raises, perhaps, more questions, and initial objections than substantial answers, we nevertheless find it useful in providing a broader context for our analysis of the historical stadiums.

## Super-Size Me

‘The new European stadia embody a historical transformation of profound significance,’ writes King (2010, p. 34). This shift involves radical reconfigurations of older stadiums, like Old Trafford in Manchester, a re-location from historical venues and locations to new, and a much closer alliance between corporate money and sports. Impressive roofs and fancy glass façades are the new signature features of football architecture, an appearance that ‘transgresses conventional notions of boundaries and space’ (Ibid: 31). King could have added overblown proportions to the list of transgressive tendencies. European football architecture in the 2000s is generally characterized by growth: grandness of scale, grandness of ambition, grandness through expansion. Audience capacities have tended to increase along with the total volume of the stadium. More functions—cinemas, restaurants, conference venues—have been added to the design in the hope of increased profitability (Wergeland, 2012a).

Expansion has seemed like the only way forwards for football clubs with a desire for success. Writing on the strategic need for iconic football stadiums a trio of engineers put it like this: ‘Due to the constraints of existing facilities and location of their current grounds, a number of clubs have been forced to consider the complete development of a new stadium’ (Aritua et al., 2008, p. 1). In order to build stadiums that are sufficiently iconic from a marketing point of view, clubs simply *had to* leave their historical facilities. This kind of development, argues sociologist Ramón Llopis-Goig, has altered the configuration of football culture: ‘During the past 20 years, European football has witnessed an intense change process that has radically transformed some of its main structural characteristics’ (Llopis-Goig, 2015, p. 104). This has led to hyper-consumption on one side and detraditionalization on the other, symbolized by the widespread urge to replace old stadiums with new.

In football architecture, size obviously matters. But instead of finding the right size, from a long-term perspective, much effort and money had been used on building as large as possible to appear attractive and impressive from the day they open. Up until recently, this approach found support in several publications on sustainable architectural design, most notably *Big & Green* (Gissen, 2003), an anthology on large-scale green architecture. While large buildings such as skyscrapers, shopping centers and apartment complexes are the worst when it comes to energy consumption and waste management, they can become more environmental-friendly if the appropriate construction systems and design strategies are employed (Gissen, 2003, p. 10–11). The advantage of large sports venues in this regard is that they can yield substantial results in terms of energy use and environmental impact. Large façades mean vast spaces for solar panels. Large roofs mean more surface for rain harvesting. If successful, it could have a huge impact on the environmental credibility of sports architecture.

On the other hand, however, size can be a significant problem. As admitted in *Big & Green*, ‘the construction of buildings is consuming some *three billion tons* of raw material every year [..]. This gargantuan appetite for raw materials results in some of the same problems associated with the production and consumption of consumer goods’ (Braungart, 2003, p. 115). The larger one builds, the bigger the problem, especially when the building is designed to host vast audiences. Sport stadiums attract more people, generate more transport, add more pressure on a piece of land and cause more consumption compared to most other buildings. As the scale goes up, more energy is needed to make the stadium operational.

The big scale also comes with a sizeable time-pressure. Huge stadiums are built to look smashing and function perfectly from their inauguration day. However, as previous studies have shown (Matheson, 2008; Wergeland, 2012b), many icons crumble in post-championship mode. Mega-event flagships mean mega-challenges, argues Deng and Poon (2017), who list overstated image building, volatile organization and pricy forgetfulness as the major reasons behind post-event dilemmas. Müller (2015) adds a few more in his dissection of the mega-event syndrome, as he calls it. One should also put maintenance issues on the list, especially in cases where the architectural design is large and complex. This adds further pressure on the daily care, which typically is a subject of neglect in large architectural projects (Sample, 2016).

Another important element is the social dimension. Football stadiums worldwide have for more than a century carried iconic significances in many communities, with stands designed to accommodate both the active and passionate fans and the more neutral spectators (Frank and Steets, 2010, p. 1–16). With an urban infrastructure providing both transport to and from the stadium and social meeting points in the shape of pubs, cafes and social clubs, football has since the turn of the last century provided its *aficionados* with rich opportunities for an extended sociality with friends and foes also before and after games. Until the more radical transformation of stadium architecture from the early 1990's stadia were generally designed to accommodate both active fans in standing terraced areas, to which it was usually cheaper to buy a ticket, along with more comfortable seated sections. Due to several incidents of ‘symbolic hatred’ evolving into violent clashes between rivaling fans during the 1970's and 80's, clubs and authorities introduced CCTV surveillance cameras and also started to ‘pen in’ sections of the stadium, meant to stop fans from both pitch invasions and clashes with rivaling fans (Armstrong, 1998). This meant that fans, once inside a stadium, would find themselves surrounded by barbed wire perimeter fences from which there were no obvious escape route in emergency situations. Some of the most serious incidents of crowd disasters in the history of the game have been caused by derelict facilities, crowd congestion and poor policing, such as in the case of the stadium disasters at Heysel in 1985, Bradford in 1985, and Hillsborough in 1989 (Darby et al., 2005). These accidents also happened as a result of dense crowds participating in the often intense social dramas unfolding during football matches. The subsequent investigations, especially the one from the disaster at Hillsborough stadium in Sheffield in 1989, lead to the so-called ‘Taylor report’ which provided a series of new guidelines regarding safety and security at football stadiums (Hillsborough Stadium Disaster Final Report, 2000). Among the more dramatic turns was the new requirement for all-seated stadiums, which meant that clubs either had to refurbish their existing stadiums with a lower crowd capacity, or choose to find land and get permission from local authorities to build a new stadium. The latter alternative meant that most clubs would have to move out of the city to a suburban or green-belt area with a weaker or non-existent social infrastructure and environmentally unsustainable modes of transport.

These planning dilemmas were of course combined with a commercial approach in which leading clubs and football authorities started to focus less on a one-sided concern for social control and more on the comfort of spectators, and thus threatening to uproot the often passionate social identities attached to a football stadium as a *topophilic* landscape, analyzed so elegantly by Bale (1991, 1993a) in the early days of these transformations. The rise of independent fanzines in Britain and elsewhere from the late 1980's and the establishment of independent supporters clubs from around 1990, should be seen as cultural grassroots responses and, in some cases, resistances, to these new ways of thinking about stadium architecture and the development of football as a spectator sport and a business (Haynes, 1995). In some cases, the concerns of fan groups have been of an existential concern as clubs started to ponder various options, with stadium relocation, sometimes with a merger with neighboring clubs, by many seen as real threats to the sociality and passions of football as they knew it.

In the three decades since 1990, most stadiums have been refurbished on the same site as the old ones were located. However, a significant number of football clubs have also opted to construct new stadiums elsewhere, usually in suburban, green-belt areas outside city centers. The provision of nothing but an open-air brick wall for urinating inside Scottish football grounds, experienced by one of the authors here in the early 1990's, is now a fading memory, with somewhat limited nostalgic potentials. However, the compact social infrastructure of that era has in many cases given way for socially more limited practices around games as suburban stadiums with greater comfort and inflated ticket prices have significantly altered the access and nature of stadium landscapes.

As Olof Moen has ascertained (1995), there is a long-standing tradition in football for the inner-city neighborhood scale, simply because most football grounds developed from open and available spaces adjacent to homes, shops and factories. This meant that the structure was relatively dense and that there were close ties between the local social life and the sports architecture. On the plus side, this led to an intertwined relationship between sports and the neighborhood, forging geographical and emotional bonds between club and community. On the minus side, it became increasingly difficult to redevelop the stadium in accordance with growing expectations with regards to capacity, comfort and logistics. This tension, argues Moen, has been the source of much debate among football fans and inner-city residents. If the scale goes up and the stadium moves to a different location, it is never just a practical operation—it signifies a change in values and ideas (ibid: 208-209). Such changes imply an infringement of existing social contracts between the stadium, its audience and its immediate neighbors.

Lack of social engagement—the super-size me approach—is also about super ‘hyper—commodification’ (Giulianotti, 2002). The emphasis on flow, comfort and efficiency has little care for crooked old streets and narrow neighborhood structures. Inner city areas have been replaced with suburban hinterland. This relates to a long-standing trend in urban sports infrastructure investment, which has primarily been aimed at tourists instead of local communities (Gratton and Henry, 2001).

## The Greening of Football Architecture

Despite the obvious problems, contemporary football architecture nevertheless prides itself with green rhetoric. A typical example is Eco Park, a new stadium project for Forest Green Rovers in Gloucestershire, England, which was granted planning permission by the Stroud District Council in late 2019. Designed by the renowned Zaha Hadid Architects, it is marketed as ‘the world's first timber stadium.’ While this marketing strategy ignores the fact that timber was the main material in the football stadiums of the late 19th and early 20th century, the Eco Park project is influenced by the technology-driven optimism of the sustainable architecture discourse of the early 2000s. This approach puts existing buildings under pressure in the name of green development. After the tragic disasters of the 1980's, mentioned above, football architecture rejected its heritage. Some of the early examples of demolition and replacement, such as Bolton Wanderer's move from Burnden Park to Reebok Stadium in 1997, did not necessarily involve eco-ambitions. But it quickly became apparent that green impulses were seeping into sports architecture. The Sidney Olympics in 2000, the so-called ‘Green Games,’ was a pivotal moment, diverting much attention to the environmental cause (Waitt, 1999; Davidson and McNeill, 2011). The 2006 FIFA World Cup in Germany sparked much of the same hype and interest in the field of football architecture (Helzel and Felix, 2006; Eick, 2011).

One reason for this optimism on behalf of sustainable technology is that the governing bodies of sports always chase the next tournament and the next building project, rather than critically assessing what they just left behind. ‘After each Olympics and the disappointing economic outcomes, the IOC puts its well-oiled propaganda machine to work,’ as Zimbalist (2017, p. 2) puts it. He could have added disappointing ecological outcomes to his critical verdict. This forward-driven approach has pushed football architecture in a direction where environmental-friendly features like water harvesting, solar panels and automatic waste management have become mandatory, often paired with planning schemes inspired by green urbanism (Radovic, 2009; Haas, 2012). The basic idea is to boost the climate capacity of a building by wrapping an eco-urbanity around it; an urban ecosystem in which buildings merge with the natural environment. The masterplan for the 2016 Rio Olympics is an example of this. Marketed as ‘A masterplan serving today and tomorrow’ with ambitions of ‘leaving a lasting legacy,’ the overriding goal was to ‘deliver sustainable Games in the very broadest sense, so the host city derives ongoing economic, social and environmental benefits’ AECOM (2012).

The challenge embedded in such visions, regardless of how eco-aware and people-friendly they may be in theory, is that the ‘green’ signaling power of mega-events fades away after the end of the tournament (Preuss, 2013). When reality hits, the legacy suffers. Therefore, ‘legacy’ must not be confused with ‘sustainability’. Legacy can also cover historical heritage but it is much broader and loosely defined as a term, and therefore ‘easily manipulated to suit different ideologies and, in the case of the Olympics, to fit into different meta-narratives of urban development.’ (Gold and Gold, 2013, p. 3527–3528). Typically, mega-events cause more physical and economic change than conservation of existing natural, cultural and social values. As Renata Sanchez and Stephen Essex put it: ‘Although world-acclaimed architectural offices were involved in the design of Rio's Olympics works, the projects seem to neglect their users. The legacy of the built environment created by the Rio Olympics appears to be counter to the creation of a sustainable, mixed-use community, and the area is poorly connected and integrated with the rest of the city.’ (Sanchez and Essex, 2017, p. 101). Contrary to good intentions, Rio 2016 did not improve the city's natural and social environment according to expectations. From a circular design point of view, events like London 2012 and Rio 2016 are doomed to fail because of the inherit mega-ness of the whole operation. Despite pre-existing plans of reduction, recycling and downscaling, it is notoriously difficult to shrink, adapt and reuse in a discourse where grandness and spectacle lies at the core.

What, then, about new and so-called ‘eco-friendly’ stadiums—are they better than the disappointing standards of Olympics and FIFA World Cups with regards to sustainability? The hype has certainly been on, especially since the 2006 World Cup in Germany, when many stadiums were either built anew or refurbished with environmental concerns in mind. One example is the Mercedes-Benz Arena in Stuttgart, an existing venue that got refurbished ahead of the World Cup. Still, the eco-boosting solutions were primarily new elements pasted onto the existing structure, like the roof, which can harvest rain water and make it reusable. If a more holistic approach had been in place, the Mercedes-Benz Arena could have qualified as an example of circular design.

The lack of a holistic approach is precisely the problem with many new stadiums. As argued by Schimmel (1995), stadium construction has been dominated by micro-perspectives: the internal factors—economy, security, logistics etc.—of the stadium design. When considered in isolation from the larger urban context, sustainable performativity is obviously much easier to achieve. The problem is, however, that stadiums must be measured against the total impact they have on the larger urban terrain in order to assess their carbon footprint and other environmental impact parameters. This means that most of the positive examples we have seen over the past years also carry problems. Some projects, like Allianz Stadium in Turin, have tackled the question of size well—Juventus have downscaled the crowd capacity compared to their previous home, the Stadio Communale—and the entire stadium façade is clad with solar panels. On the macro level, there are however at least two highly significant factors that undermine the focus on renewable energy and downscaling: Firstly, the stadium combines a massive car park with no substantial solution for public transit, which means that the stadium generates private car use as the major mode of transport. Secondly, the stadium is situated in a desolate area of the city. This kind of location is typical for the suburbanization trend of the 1990s (Bale, 1993b; Horak, 1995), which saw football clubs all over Europe moving from the city center to the urban fringe. This form of development is completely at odds with the compact city model commonly associated with sustainable urbanism today (De Roo and Miller, 2019). Allianz Stadium has already been criticized for its failure to comply with this principle (Lekakis, 2018).

The relocation strategy also carries negative social consequences, as further detailed in our study of Tynecastle Park in Edinburgh below. New stadiums are often portrayed as a gift to the local community but they repeatedly fail to deliver genuine local qualities. This is particularly evident in the aftermath of mega-events (Jennings and Lodge, 2011; Kowalska, 2017; Zimbalist, 2017; Dendura, 2019). While the geographical catchment area of football has increased dramatically over the past decades, the significance of the inner city and a central location has never faded in the minds of local stakeholders (Bale, 2003, p. 84–107). A football supporter community engages with its team, the local context, and the network of local actors in multiple ways. Their passion, excitement, and involvement plays a crucial role in the production of cultural and economic values in and around the club; an intricate process of co-creation which is often overlooked by owners and investors who are keen to relocate (Zagnoli and Radicchi, 2013). The rational reasons for moving must therefore be carefully considered against a dominant desire to stay if football clubs wish to also remain socially sustainable. Even in cases when big clubs remain in the same area, exemplified by Arsenal's love from Highbury to Emirates Stadium, there may be unresolved issues from a sustainable heritage point of view. Very little of the building mass of old Highbury exists today, and the social culture in the new stadium is completely different due to sky-rocketing ticket prices and other factors which exclude traditional local supporters.

With all these obstacles in mind, it seems almost impossible to imagine that football architecture will become more sustainable in the foreseeable future. On the plus side, there have never been more theoretical and practical solutions at hand. If we look to the avant-garde of contemporary preservation and architecture, two concepts immediately emerge as the frontrunners, inspired by circular thinking: Adaptive reuse and maintenance architecture. Adaptive reuse is essentially aimed at combining preservation techniques with alteration and modernization. This can be based on a variety of different traditions, from careful restoration to progressive transformation (Plevoets and van Cleempoel, 2019, p. 7–27). It is a diverse theoretical term that offers a range of practical solutions and intervention strategies (ibid: 28–51). It can be applied to all sorts of buildings, ancient and modern, small and large.

Maintenance architecture mainly comes from the book of that title (Sample, 2016), but the concept is now spreading across the field of architecture through the use of words like ‘repair’ (Baracco and Louise, 2018) and ‘fixing’ (Livas, 2019) in book titles. ‘Maintenance plays a crucial role in the production of architecture, yet by and large architects have treated it with indifference’, claims Sample (2016, p. 1), with reference to how the modern architectural discourse has created a cult of worship around buildings that are new or appear to be new. This has created ‘falsely constrained endpoints—conception and realization’ (ibid: 7), which means that architecture is often judged as eternally new-born monuments—an impossible condition for any building as decay inevitably sets in. Architecture, she argues, must begin to appreciate the professions that secure permanence, endurance, and preservation of buildings. The building industry needs more input from caretakers and janitors. Sample refers to the massive job of cleaning Beijing's National Aquatic Center, built for the 2008 Olympics, as a lesson to learn from (ibid: 155). If you erect large sports venues in a highly polluted city, you have to tackle the consequences.

## Case Studies

In this section we outline the key findings and perspectives from our recurrent field work and research on Tynecastle Park and Stadio Flaminio in light of the principles for reuse, maintenance and circular heritage presented above. Each study provides an overview of the history of each stadium, emphasizing major events and turning points, and an assessment of their current standing. Our study of Tynecastle park deals mostly with the issue of social sustainability in light of the stepwise transformation of an historical football arena. Our study of Stadia Flaminio is primarily concerned with the technical aspects of sustainability and the challenge of re-opening an abandoned venue.

### Tynecastle Park

‘Fans’ route to the stadium involves a bodily involvement with the materiality of the environs, producing placed experiences of heartbeat and breath, the particular movement of limbs and the sensing of textures underfoot, the press of bodies, the assailing of the nostrils by familiar smells, and the sonic melding of one's own footsteps with those of a thousand others' (Edensor and Millington, 2010, p. 155).

**Figure 3:**
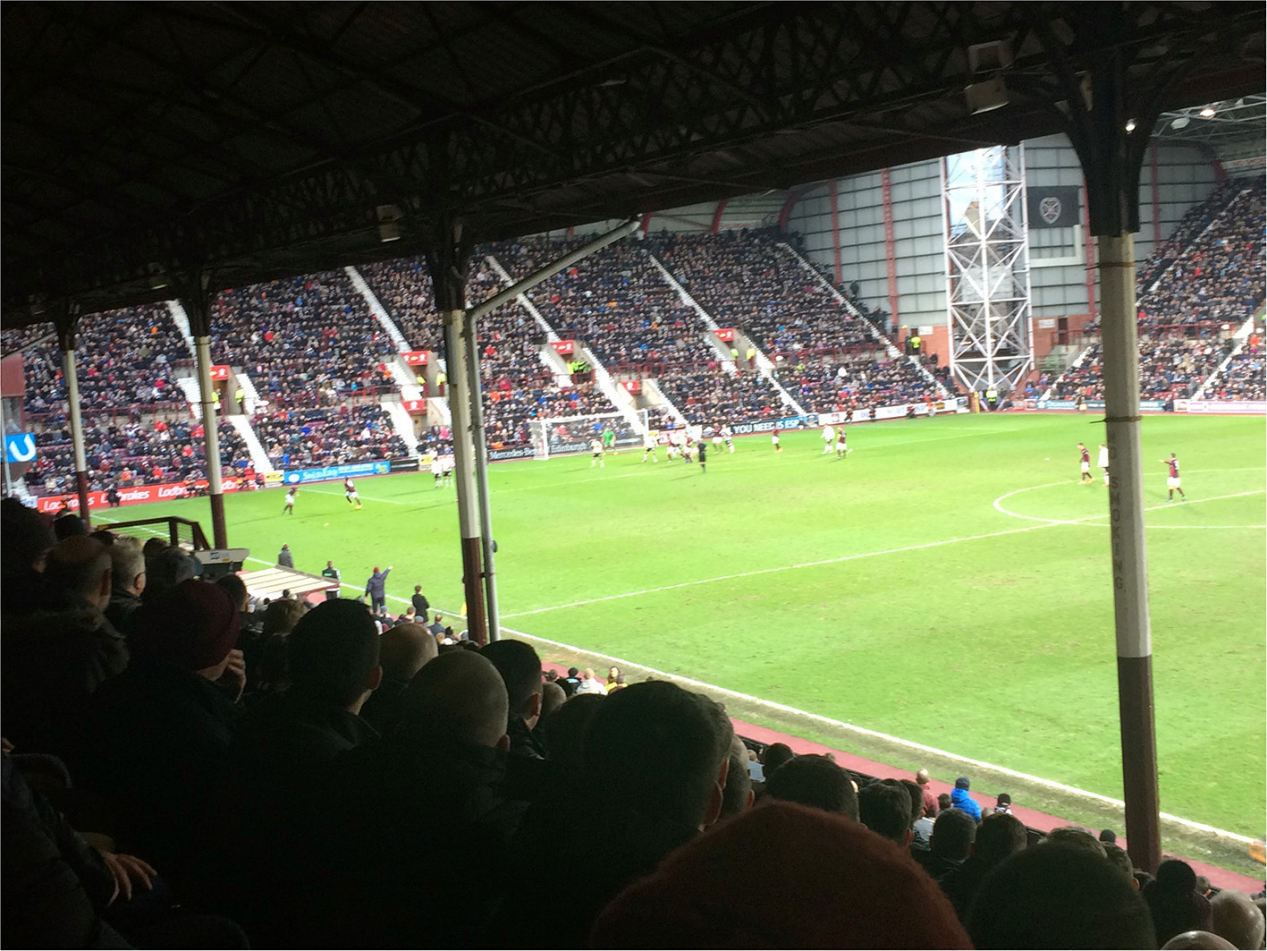
Old meets new–view from the old main stand at Tynecastle Park, built in 1914 and demolished in 2017. The Gorgie Road end and Wheatfield stand, erected in 1996, are visible in the background (Credit: Hans K. Hognestad).

Tynecastle Park is the home of the Scottish professional football club Heart of Midlothian. It is located in Gorgie, a short walk west of the Edinburgh city center. Tynecastle Park (Figure 3) may be described as an archetypical British football ground due to its urban location. Since the early 1900's the stadium has been surrounded by a school, a whiskey distillery, a church and tenements. While required substantial refurbishments have taken place in recent times, the club have had their home in Gorgie since 1881 and the original construction was part of a wider urban development of the area at the time. A new main stand, designed by the famous stadium architect Archibald Leitch (Inglis, 2005), was built in 1914 while the rest of the stadium was refurbished and gradually expanded in subsequent decades to hold crowds of up to 50.000 (the record attendance of 53,396 from 1932 still stands). After World War II old wooden terracings were replaced by concrete steps, making Tynecastle the first all-concrete stadium in Scotland in 1954. Perimeter fences were put up in the 1970's as a crowd control measurement, while a previous standing area along one of the sides was made into a seated area, cutting the capacity to 29.000 by 1981. Six years later the stadium author Simon Inglis viewed Tynecastle Park in the following way: ‘Many British football grounds are hidden in cramped inner-city locations, but none, surely are as penned in as Tynecastle. Tenements and a sootcoloured distillery watch over the ground like cell-blocks over a prison yard, while the stands are clothed in a brooding, dark maroon; the maroon of old British railway stations and also of Edinburgh buses. Hearts-fans must once have felt very much at home on their travels... It all adds up to an inner-city *melange* which however inconvenient or outdated, few would wish to change one little bit. Just as tourists delight in the ramparts and dungeons of Edinburgh castle, so too do lovers of Scottish football delight in the cloistered intricacies of Tynecastle’ (Inglis, 1987, p. 338–9). However, change was indeed looming by the early 1990's.

**Figure 4:**
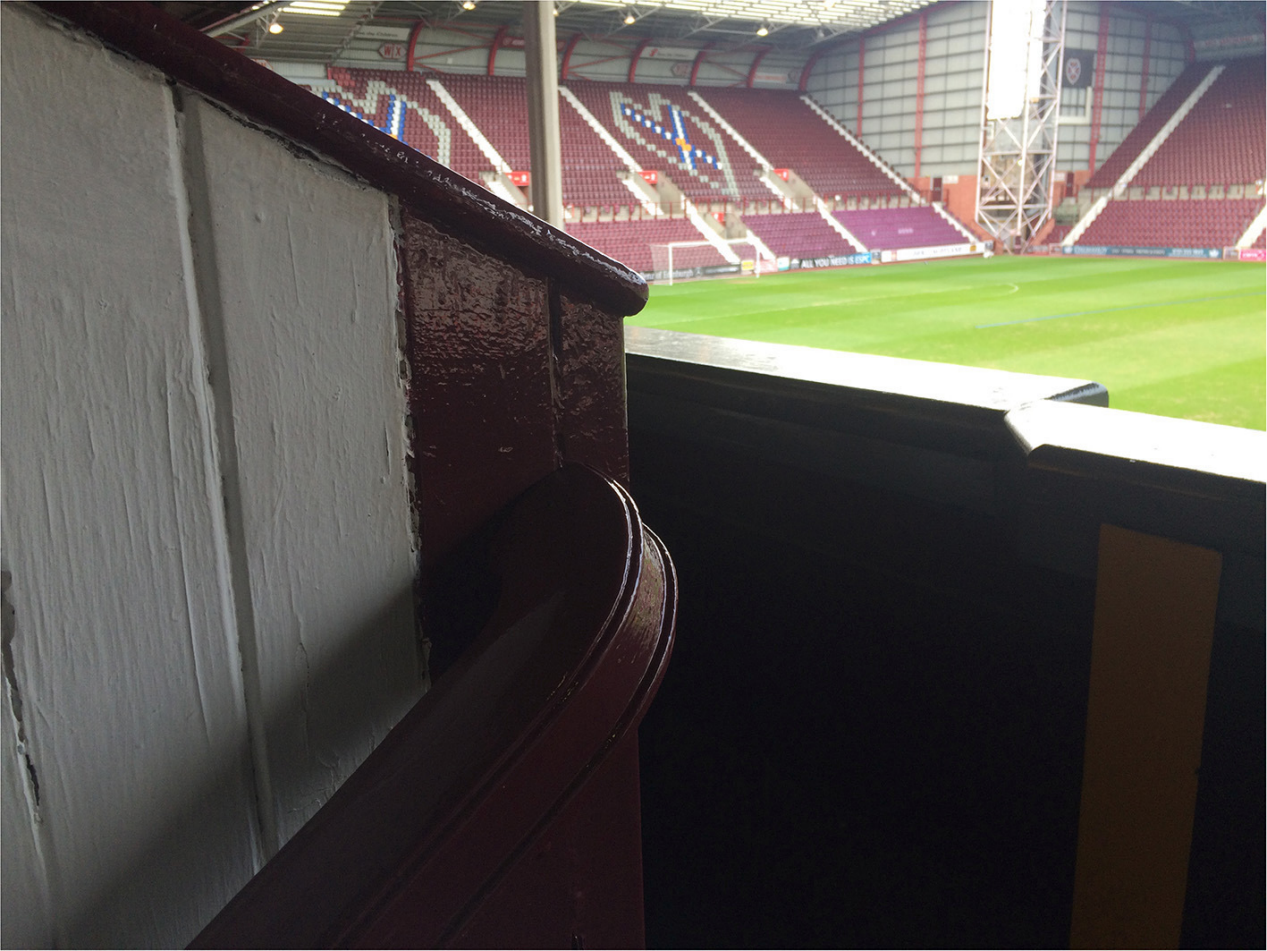
Detail from the wooden railing at the old main stand at Tynecastle Park (Credit: Hans K. Hognestad).

In order to meet the recommendations outlined in the Taylor report after the Hillsborough disaster in 1989 (Hillsborough Stadium Disaster Final Report, 2000), the club was faced with a few soul-searching decisions which led the chairman of the Hearts at the time, Wallace Mercer, to come up with proposals which generated substantial resistance and indeed fan activism. Mercer announced in May 1990 that he had entered an agreement with David Murray, a well-known Scottish business-entrepreneur and owner of Rangers Football Club at that time, to develop a new 25.000 all-seater stadium as an element of a huge business-project Murray was planning at Millerhill, a green belt site located east of Edinburgh's city center. The idea was to incorporate other facilities like greyhound tracks, a diversity of community-facilities and make the stadium into a potential venue for pop concerts (Moorhouse, 1991, p. 216). The club was hoping to have their new, ‘multi-purpose’ stadium ready by the 1993/94 season. This proposal gave the uprise to heated debates in various media by anyone who had strong feelings connected to the club and its location in Gorgie. The makers of a fanzine called *Dead Ball*, expressed their views on the re-location plans and a move away from ‘Tynie’ (a local nickname for Tynecastle Park) like this: ‘Any move from Tynecastle to a custom-built stadium is not just a change of location for a football team. It is also in a real sense a violent act. To destroy a central part of many people's lives, whilst it may be progress of sorts, is to put imagination after money. Tynie is not just a football ground; it is part of me, part of us all. It is a concrete representation of the club's history. It wasn't built just with bricks, mortar and wood, it also took dreams, hope and (mostly) despair. It represents the soul of Hearts. The memories of past greats still inhabit the place. It is a connection to Walker, Mackay, Bauld, Conn, Waurdaugh and Ford [former players at the club]... It almost doesn't matter that Tynecastle is a dump’ (*Dead Ball*, no 1) (Figure 4).

Both of these views present the link between the club, its location and its fans as an insoluble unity. And the very existence of the club is, according to these views, tied to the ground of Gorgie, West-Edinburgh. Even though most fans had a strong feeling for the place, there were few people who were against change as such, in the way Inglis' romantic description, quoted earlier, seems to suggest. As the last ironic comment in this quote from *Dead Ball*, indicates, there was also little sentimentalism attached to the technical standard of the ground in the early 1990's. They opposed any move away from Tynecastle and Gorgie, but desired a functional and more modern stadium that could meet the necessary requirements as the home of one of Scotland's elite football clubs. At the time, football spectators tended to see the ground as both outdated and decaying, which seemed to alienate visiting supporters in particular, as this fan of Dundee Utd wrote in 1993: ‘Consider, also, the dilapidated Gorgie Road entrance which, set back from the road, makes visiting fans feel as though they are entering some Dickensian establishment and, once inside, the high perimeter fences which, when the ground is filled with humanity, give the place the look of a POW camp. Added to all this there is the pungent and nauseating smell of brewing that hangs over this part of Edinburgh to add to the overall ambience. I will not dwell on the toilets, no-one should!’ (McIlroy, 1993, p. 120).

A new all-seated stadium would bring an end to the much beloved intimacy of the standing terraces, but were generally preferred to an alternative multipurpose-stadium out of the city. The belief that Tynecastle Park, Gorgie, is the home of Hearts, was prevalent and represented fans' identification with the club in a concrete way. This must be linked to the ways in which ‘the people’ have filled a place provided by an industry with commercial interests, with its own meanings: ‘The landlord provides the building within which we dwell, the department store our means of furnishing it... But in dwelling in the landlord's place, we make it into our space; the practices of dwelling are ours, not his.’ (Fiske, 1989, p. 33). What is peculiar here, is the fact that the whole construction of what Zukin calls the ‘vernacular space’ (Zukin, 1992, p. 224) was literally threatened by removal. For a lot of fans this meant a threat to the very existence of the community which had grown into the walls and terraces of Tynecastle Park. The proposed move away from Tynecastle would have included more than a relocation of a football-ground. The urban infrastructure around Tynecastle Park, in the shape of numerous pubs and social clubs easily accessible by bus or foot, were equally important to the total football experience for a lot of fans: ‘I mean we love Gorgie and Tynecastle. It's a great area. The problem with new all-seated stadia outside the city or town is that you've got no pubs to go to before the game. You go to Perth and their new stadium is great, but you cannot go for a pint before the game, apart from one which is absolutely mobbed. At Tynecastle you've got maybe 20 to 30 pubs within a 5 min walk from the ground, so you've got a choice and each pub has got its own character, its own history as well' (Peter, 38, personal interview March 1993). The significance of the historical social stability on match days provided by the many pubs and social clubs in the Gorgie area should not be underestimated. One of the authors here were once shown a booklet with fixtures for Hearts games for the 1895–96 season by a collector of football memorabilia. At the end of this booklet there were several advertisements, one of them for ‘The Midlothian Arms,’ which was the old name for Tynecastle Arms, a pub still located right next to the stadium, on the corner of McLeod Street and Gorgie Road. The advertisement read: ‘Before and after games, enjoy our fine selection of wines, spirits and ales.’ With the proposal to move away from its historical home, it was generally feared that this century-old sociality of football would be displaced and limited to the act of taking your car to watch games in Suburbia.

Jim Clydesdale, a director at Hearts F.C. at the time and also an architect, presented the club's vision of a new multipurpose-stadium at Millerhill in a more detailed way, at a seminar held by the Scottish Sports Council inMarch, 1991. The following quotation is taken from the fanzine *Always the Bridesmaid* (1991), who put their own heading above the reprinted abstract of Clydesdale's paper: ‘Cloud Cuckoo Land,’ meant to illustrate the lack of touch with ‘the ordinary supporter’ that this vision demonstrated. The outline was clealry aimed toward facilitating the tastes of a middle-class family with more money to spend, an attempt perhaps to invite spectators more akin to the *flaneur* category outlined in Richard Giulianotti's football spectator taxonomy a decade later (Giulianotti, 2002). *Dead Ball*, the fanzine quoted earlier, described this multi-purpose concept as a ‘post-modern nightmare,’ referring to the multitude of ‘leisure-activities’ this stadium plan would hold (personal interview, October 1992). However, Hearts-supporters were joined by environmental activists who protested against further exploitation of greenbelt land outside the city center, while members of the Lothian Regional Council also objected against the proposal (Moorhouse, 1991:216). In the end too many voices were raised against an approval of these development-plans, and the Lothian Regional Council turned the proposition down in August, 1992. Hearts F.C. had to come up with other solutions. After looking at a few other locations, the club finally announced in December 1992 that they were in fact planning to redevelop Tynecastle into a smaller all-seated stadium. However, it wasn't until a year later that concrete plans were made for redeveloping Tynecastle Park. A proposal from the Lothian Regional Council that Hearts could share a new ground with local rivals Hibernian Football Club, who had received permission for building a new stadium at Straiton, 20 km south of the city center, was rejected by Hearts in October 1993, following considerable activism from both sets of supporters. In the end even Hibernian decided to redevelop their old ground, Easter Road in the Leith area, just east of the Edinburgh city center.

Three of the stands at Tynecastle Park were demolished and replaced by new concrete stands between 1994 and 1997. The new all-seated stadium capacity was now reduced to 17,500, which was nevertheless generally seen as preferable to a new and bigger stadium in a greenbelt area outside the city. Eventually the last remaining main stand from 1914, was demolished in 2017. The new main stand, opened in late 2017, increasing the capacity again to almost 20,000. The stands built in the 1990's and the one in 2017 were all designed by the same architect, Jim Clydesdale, quoted above with a totally different stadium vision. Yet, during the 20 years between 1997 and 2017 there were further attempts made by the board at Hearts F. C. to leave Tynecastle Park, in favor of a bigger venue. In 2004 the then chairman of the club, Chris Robinson, had announced that the club was ready to sell Tynecastle Park for other purposes and rent the neighboring national rugby stadium, Murrayfield, with a capacity of 67,000 instead. But once again, it was the chairman who had to leave instead, as supporters were heavily in favor of remaining at Tynecastle. The story about Tynecastle Park shows the strength and sentiments which may be attached to the location and the area, perhaps even more than to the quality of the stadium itself. While Bale (1991) has highlighted the symbolic significance of football stadiums as concrete symbolic representations of communities, a lot of such qualities are tied to the fact that the stadium is part of an urban landscape with plenty of social meeting points and easy access by foot, bike or public transport.

### Stadio Flaminio

**Figure 5:**
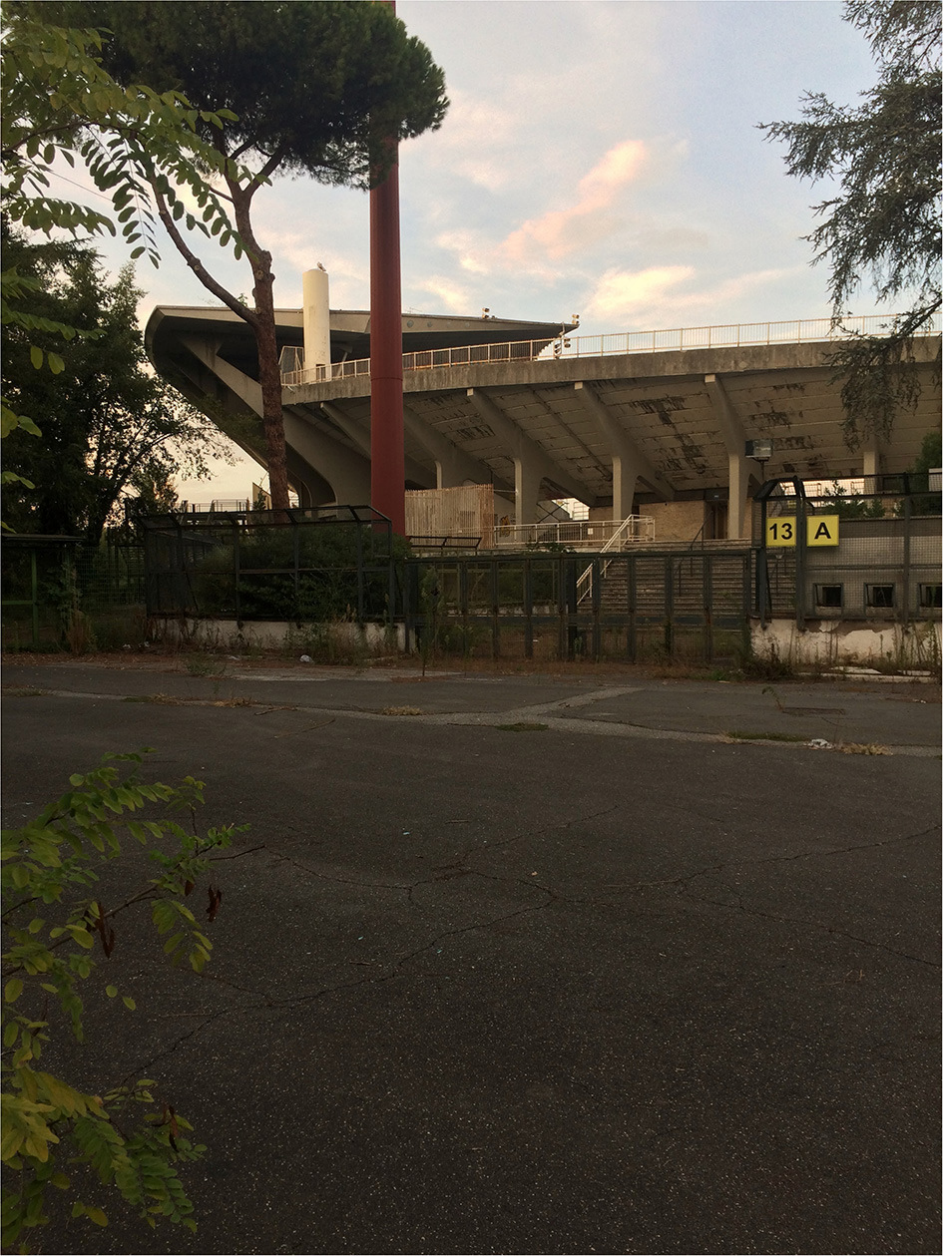
Faded elegance: Pier Luigi Nervi's concrete design in a state of advanced decay, September 2017 (Credit: Even Smith Wergeland).

The origins of Stadio Flaminio were not particularly promising from a circular heritage point of view. It was built on the same piece of land as Stadio Nazionale stood from 1911 until it got demolished in 1957, when it was deemed ‘unfit for use due to the ravages of time’ (The Organizing Committee, 1960, p. 58) ahead of the 1960 Rome Olympics. It therefore had to be replaced ‘by an ultramodern stadium’ (ibid), according to the organizing committee. We can here clearly recognize the rhetoric from today's Olympic extravaganza as well as Stadio Flaminio's own destiny in the 2010s. It's predecessor, Stadio Nazionale, was used during the 1934 FIFA World Cup and had a strong connection to the Mussolini regime, the main political force behind the development of what would eventually become Rome's Olympic Village in 1960. The political connotations may have furthered the demolition cause (Figure 5).

Stadio Flaminio is situated in Rome's Parioli district, north of the city center. The stadium is located along the Via Flaminia close to the left bank of the Tiber in close proximity to other Olympic facilities from 1960, like Palazzetto dello Sport and Villaggio Olimpico (the athlete's village). Just across the river, northwest of the athlete's village, lies Foro Italico, the main hub for sports facilities in Rome, including the majestic Stadio Olimpico. It is no exaggeration to say that this part of the eternal city is characterized by sports heritage.

The task of building a modern stadium on a historical spot was handed to Pier Luigi Nervi, the Italian engineer best known for his pioneering use of reinforced concrete as a structural and decorative material in the post-war period (Iori and Poretti, 2019, p. 3). His son, Antonio Nervi, was also assigned to the project as the lead architect. Construction went on from 1957 to 1958 and the stadium was inaugurated in 1959, a good year ahead of the Olympics, without exceeding the estimated cost. For such a large venue that is a commendable feat. Upon completion, the city of Rome had gained a 42.000 capacity arena with a number of design innovations. All sectors of the stadium were provided with bars and other services. The most striking feature from a visual point of view was the hovering roof canopy that kept about 8.000 seats under cover while it was still in use.

Although the stadium was purpose-built for football, which it hosted during the Olympics, it also featured four gymnasiums, a fencing hall, a covered and heated swimming pool, changing rooms and a first aid station. This kind of functional and spatial diversity was unusual compared to the expected standard in the post-war period. From a structural engineering perspective, the stadium was top notch. The building system, based on a complex combination of *in-situ* concrete and prefab concrete, was unique for this particular stadium. While concrete architecture is often associated with fixed modular systems and standard construction schemes, very little of the kind was used here. Instead, father and son Nervi devised a concept in which every building part—the grandstands, the structural frames, the roof canopies—had its own signature, construction-wise and aesthetically. There are similar stadiums from this period but if you study the details, the individuality of Flaminio is striking, down to the smallest nuts and bolts. This made it stand out in its prime time. It also means that the building is uniquely difficult to manage today, when entrepreneurial construction normally depends on defined standards (Sample, 2016). The brief of renovating it is therefore a bit of a challenge, which we shall explore soon.

The Flaminio was regarded a success during the Olympics and it had, for quite a long while, a purposeful afterlife. Since the Olympics, it has been used as a concert venue (both Pink Floyd, Bruce Springsteen and Michael Jackson performed there in the late 1980s), the Italian Rugby Union used it to host the Six Nations Tournament (2000–2011) and the two major football clubs of the city, AS Roma and SS Lazio, used it as a temporary venue during the renovation of Stadio Olimpico before the FIFA World Cup in 1990. It was also a home ground for Atletico Roma FC, who played in the Italian Serie C from 2005 to 2011, when the club dissolved. This event turned out to be a twist of fate for the Flaminio too, since the stadium now became vacant as a consequence and has been abandoned as a venue for sporting and cultural activities ever since.

The stadium has not been left entirely to its devices, however. A multi-disciplinary team of preservation experts, led by the Department of Structural and Geotechnical Engineering at the Sapienza University of Rome, joined forces with the Pier Luigi Nervi Project and Docomomo Italy to apply for funding within the Getty Foundation's *Keeping it Modern* programme. This application was successful and the project officially obtained the support of the Getty Foundation in June 2017 (Stadio Flaminio, 2021) to develop a four-step conservation plan under the leadership of the aforementioned Romeo. The Getty grant was awarded the same year to the Japan Sport Council in support of the renovation of Yoyogi National Gymnasium in Tokyo, built by the Japanese architect Kenzo Tange ahead of the 1964 Olympics. Suddenly, there were two sports arenas under the Getty Foundation's ‘Keeping it modern’ umbrella. Someone must have envisioned a future for old arenas. In July 2018 the Flaminio got listed as a cultural heritage site by the city of Rome, which means that it now has legal protection and formal status as culturally significant.

Even with funding and listing in place, the challenge is still pretty immense for Romeo and his team. ‘The stadium is now in an advanced state of decay’ (Stadio Flaminio, 2021) as they had to admit before work commenced. One of the biggest issues to solve is Nervi's strong dependence on concrete—a building material that still dominates in today's sports architecture. During the post-war period, Nervi's heydays, concrete became the most widely used material in the construction industry. It was cheap, efficient, accessible and flexible. It also turned out to be one of the most toxic and wasteful materials on the planet. For every ton of cement produced, approximately one ton of CO^2^ is released (Mehta and Monteiro, 2014). It devours raw materials like few other substances in the building industry. Its relation to water alone is highly problematic: concrete consumes water like a swamp, thus ‘stealing’ it from living creatures. Clearly, the hegemony of concrete in sports architecture has to be challenged in the name of sustainability. The problem is, however, that demolition of concrete architecture is also an environmental threat. The process is time-consuming, money-consuming and adds to the already negative pollution and waste account. To tear down large concrete buildings therefore makes little sense from a circular perspective. They ought to stay in use for as long as possible.

On the plus side, the technical, structural and material quality of the original work is very high compared to other concrete buildings from the same period (Romeo et al., 2021). This makes the rehabilitation all the more worthwhile. Much like the contemporary team who is now trying to fix it, Nervi surrounded himself with the best available expertise of the day during the construction of the Flaminio. Another positive aspect is the size, which makes it possible to imagine that it could work as a contemporary arena from a capacity point of view. For security reasons and the all-seating principle, it can probably only house about 30.000 spectators within the existing regulations—but that is still fairly large. If a more adaptive approach had been possible the stadium could probably reach an audience of around 40,000. This points to a crucial dilemma at sports governance level—the difficulty of operating outside the so-called ‘technical manuals’ (Dunne, 2007) of IOC, FIFA and other transnational sporting bodies. These guidelines, which must be followed in order to get international approval, can be a real obstacle for innovative preservation and adaption to local needs. This is probably going to be one of the toughest hurdles to pass for the Flaminio conservation team.

This means that a lot of effort must be placed on the fourth step of the conservation plan, which deals with guidelines for recovery and reuse. Romeo's team is undoubtedly well equipped to tackle that which lies before—the historical study, the structural analysis, the physical changes and transformations—but they have to come up with something truly remarkable in order to breed new sporting life into Stadio Flaminio. This is particularly tricky since AS Roma are already planning a stadium elsewhere in Rome and SS Lazio dream of doing the same—they have certainly been reluctant to consider a return to Stadio Flaminio (Stadium Business, 2019). What could become an option is to turn it into the official home for Italian rugby. Plans are currently under way for a three-step renovation of the entire Villaggio Olimpico. The third phase of this process is supposed to allow the stadium to return to its former glory as a major rugby venue—‘to give new life to an architectural jewel that has been left to itself’ (Stadium Business, 2018) according to Daniele Frongia, the City of Rome's Councilor for Sports. The vision has allegedly been backed by a full-bodied proposal from CONI (the Italian Olympic Committee) and FIR (the Italian Rugby Federation). Only the future will reveal whether this ‘Casa del Rugby’ (Wanted in Rome, 2018) will actually materialize but the idea seems to be well-supported by the those who matter: the city administration, the politicians of Rome and the national sports associations.

## The Rise of the Reused Stadium?

In conclusion, let us return to our opening questions in light of the two cases. Is there a future for historical stadiums? Does football architecture have a circular design potential? The most accurate answer we can give at this point is: it depends. From an overarching perspective, the following conditions are most important: Firstly, the world of sports must become willing to take better care of its architectural legacy. This will necessitate a major change of attitude, involving more reverie for the quality of historical venues, more investment in maintenance and more concern for local stakeholders. Secondly, there is dire need for legislative changes to make it easier to sustain existing sports venues as part of a local sporting culture. This means that guidelines for capacity, security and logistics must be adapted to the buildings and neighborhoods in question, not the other way around. If the current guidelines continue to apply, regardless of context, reuse will remain very difficult. Thirdly, there is need for further research and investment in pilot projects like the Stadio Flaminio restoration. Presently, there are more examples of stadiums surviving against the odds—they are typically set for demolition, but the process has been stalled for various reasons—than stadiums that survive because they are actively maintained and developed. If we get to a stage where more existing stadiums are properly financed and managed, there would be more lessons to learn from and—hopefully—positive experiences. This could become a counterweight to the prevailing approach of demolition and construction.

Based on the insights gained from Tynecastle Park and Stadio Flaminio, we would argue that there is a potential for reuse and, as a radical extension of that, circular management of football architecture. Obviously, our cases are not entirely comparable, since they represent different historical origins, different sporting contexts, different local communities, different stages of restoration, and different degrees of current usability. They nevertheless offer a number of cues for future development which could serve as a starting point for a more universal and transferable strategy for reuse of historical stadiums. From Tynecastle, there is the vital social culture, local stakeholder engagement and continuous use of the same urban property to build upon. From Flaminio, there is the technically advanced restoration, the multi-disciplinary approach, the funding and the overall plans for new use to draw inspiration from. While none of these examples can aspire to be called circular heritage in the strictest meaning of the term—too much building mass has been removed from both locations over the years without any kind of recycling—there are aspects of both that could be ‘Frankensteined’ into a fully circular model.

One thing is certain: If international sports federations and clubs want to commit themselves more to green values and withstand the test of circular principles, the journey is going to be hard, difficult and frustrating. The replacement of ‘new, large and spectacular’ with ‘durable, modest and simple’ is going to demand a U-turn of unforeseen magnitude and, probably, a new generation of sports leadership. The uplifting thing, as we have shown, is that there are theories, principles and expertise ready to aid such development. Anything can be repaired these days. If Hillary Sample is right, the architecture of the future will be less about conception and realization and more about durability: ‘An expanded building cycle that incorporates maintenance has the potential to affect the future of architecture contributing to the cycle of creation, building, occupancy, the representation of architecture, and image circulation, which in turn will impact invention.’ (Sample, 2016, p. 9). Through this surprising turn of events, the old school of preservationists has re-emerged as the avant-garde. The unlikely rise of the reuse stadium represents a similar chance of re-branding football culture.

## Author Contributions

All authors listed have made a substantial, direct and intellectual contribution to the work, and approved it for publication.

## Conflict of Interest

The authors declare that the research was conducted in the absence of any commercial or financial relationships that could be construed as a potential conflict of interest.

## Publisher's Note

All claims expressed in this article are solely those of the authors and do not necessarily represent those of their affiliated organizations, or those of the publisher, the editors and the reviewers. Any product that may be evaluated in this article, or claim that may be made by its manufacturer, is not guaranteed or endorsed by the publisher.
